# Use of home visits increases data completion and retention in studies involving family members

**DOI:** 10.1186/1745-6215-16-S2-O24

**Published:** 2015-11-16

**Authors:** Katie Biggs, Cindy Cooper

**Affiliations:** 1 University of Sheffield, Sheffield, UK

## Background

Previous research indicates that health and well-being is adversely affected in family members of a child with a health condition but the practical aspects of recruiting, retaining and collecting data from family members are not well reported.

Using data from a cross-sectional observational study, we looked at whether use of home visits had an effect on the data completion and retention of family members.

## Methods

Various recruitment and data collection methods were used in the study including an option to conduct home visits with the families.

549 families living with a child with ADHD, and 123 families without any children with ADHD (control) were recruited.

## Results

When a home visit was conducted 84% of questionnaires were completed, compared to 59% when no home visit was conducted (Χ2=51.194, df=1, p<0.00).

A binomial logistic regression model included home visit, presence of ADHD and employment as significant variables, though the effect of employment was negligible.

In regards to data completion, a home visit increased the likelihood of completing the primary outcome. When a home visit was conducted 96% of children completed the CHU-9, compared to 90% without a visit (Χ2=12.300, df=1, p<0.00) and 85% of adults completed the EQ5D with a home visit, compared to 69% without (Χ2=37.038, df=1, p<0.00).

## Discussion

Retention and data completion was better in families that received a home visit, particularly in families where there was a child with ADHD.

